# Covered Socket Residuum (CSR) in Former Third Molar Sockets Despite Platelet-Rich Fibrin: A Prospective Randomized Controlled Split-Mouth Clinical Study

**DOI:** 10.3390/bioengineering12111242

**Published:** 2025-11-12

**Authors:** Shahram Ghanaati, Atoullo Kamolov, Jerry Bouquot, Robert Sader, Anja Heselich, Sarah Al-Maawi

**Affiliations:** 1 FORM-Lab, Department for Oral, Cranio-Maxillofacial and Facial Plastic Surgery, Medical Center of the Goethe University Frankfurt, Goethe University, 60590 Frankfurt am Main, Germany; atoullo.kamalov.1988@gmail.com (A.K.); r.sader@em.uni-frankfurt.de (R.S.); heselich@med.uni-frankfurt.de (A.H.); sarah.al-maawi@unimedizin-ffm.de (S.A.-M.); 2 Department Oral & Maxillofacial Surgery, West Virginia University, Morgantown, WV 26506, USA; bouquot@aol.com

**Keywords:** third molar sockets, PRF, bone substitute materials, covered socket residuum, CSR, socket preservation

## Abstract

Third molar extraction is a common oral surgical procedure that can be accompanied by challenges in wound healing and bone regeneration. Materials such as bone substitute materials (BSMs) and platelet-rich fibrin (PRF) are often used to support socket regeneration. This prospective randomized controlled split-mouth clinical trial compared PRF application combined with BSM versus PRF alone in patients requiring bilateral third molar extraction. A total of 15 patients underwent standardized osteotomy procedures, with sockets filled either with PRF alone (control group) or with BSM + PRF on opposite sides (test group) under general anesthesia and with patients blinded to the treatment allocation. Postoperative pain and swelling were measured over 7 days using a visual analog scale and anatomical distance measurements, respectively. Bone regeneration was evaluated using cone beam computed tomography (CBCT) scans after an average healing period of six months, with results showing no significant differences between groups in terms of postoperative pain or swelling (n = 12; 3 patients were lost to randomization). However, CBCT imaging revealed covered socket residuum (CSR)—non-mineralized areas within the socket—in the PRF only group, whereas the BSM + PRF group demonstrated more homogeneous and mineralized bone formation throughout the extraction sites (n = 8; 5 patients were lost to follow-up). These non-mineralized areas represent covered socket residuum within the extraction sockets, which poses a clinical risk of infection and may negatively affect the dental health of the adjacent second molar. Based on the presented findings, we recommend combining BSM with PRF to support bone regeneration and regulate the postoperative pain and swelling following third molar extraction. Nevertheless, further research is required to determine the most suitable BSM type in this regard.

## 1. Introduction

Defined by their abnormal eruption path or displacement within the jaw, ectopic third molars represent a relatively common clinical finding [1]. The condition is often associated with insufficient space in the dental arch or abnormal angulation, leading to disturbed eruption patterns, particularly in young patients [2]. It may result in symptoms such as pain, swelling, dental caries, infections, or abscesses [3].

Third molar removal can generally be classified into two categories: prophylactic and therapeutic indications [4]. The former are primarily based on insufficient space within the jaw and may be related to orthopedic and orthognathic treatments [5]. Additionally, third molars are prophylactically removed when there is a significant risk of caries affecting adjacent teeth or a general risk of infection—for example, prior to chemotherapy, immunotherapy, or planned pregnancy. The need and appropriate time point for prophylactic removal vary according to the individual patient’s condition and considering perioperative risks, especially unavoidable intraoperative damage to adjacent structures such as the lower alveolar nerve, the maxillary sinus or adjacent teeth [4,6]. Currently, prophylactic indications for third molar removal remain a subject of ongoing debate within the scientific and clinical literature [5]. In contrast, therapeutic indications are largely more urgent due to symptoms and patient discomfort in terms of symptomatic pericoronitis, recurrent infections, abscess formation, periodontal disease or tooth-associated cystic lesions [7,8].

In addition to the intraoperative risks, a number of relevant postoperative complications may arise. Among these, early- and delayed-onset infections are the most common. The former typically occur within 7 to 10 days after surgery and are characterized by swelling, wound healing disorders, and purulent discharge. These infections have been extensively analyzed, with risk factors including patient compliance, intraoral hygiene, and pathogenic contamination having been identified [9]. In contrast, delayed-onset infections usually emerge around one month postoperatively and in some cases may even occur several years after extraction, with an incidence ranging from approximately 0.4% to 9.2% [10]. The etiology of delayed infections remains unclear, although some studies suggest contributing factors such as surgical procedure complexity, perioperative medication, and overall patient health status [10]. Various medical interventions have been investigated with the aim of reducing postoperative complications and promoting socket healing. For example, piezo-surgery, low-level diode laser therapy, and photobiomodulation have shown promising results [11,12]. Additionally, platelet-rich fibrin (PRF), a blood concentrate obtained from the patient’s own peripheral blood, is widely used to support wound healing in oral and maxillofacial surgery. In this context, regenerative components of peripheral blood are concentrated via centrifugation, resulting in a fibrin matrix enriched with platelets, leukocytes, and their associated growth factors [13]. Numerous studies have highlighted the potential of PRF in enhancing wound healing, particularly in intraoral applications. However, evidence regarding PRF’s capacity to support bone regeneration remains controversial [14,15]. A recent study conducted by our research group demonstrated that, following premolar extraction, covered socket residuum, i.e., non-mineralized area within the bone, was detected regardless of PRF application [16].

Moreover, bone substitute materials (BSMs) are widely used to treat tooth extraction sockets supporting bone regeneration [17]. This intervention is usually applied as a pre-implantologic procedure, when dental implants are planned. However, supporting bone regeneration may also be necessary in third molar extraction sockets to prevent delayed onset complications and the formation of such covered socket residuum [18].

Therefore, the aim of the present randomized controlled clinical study was to evaluate the efficacy of BSM combined with PRF (test group) compared to PRF alone (control group) in supporting post-extraction socket healing following third molar removal. The null hypothesis stated that the application of BSM with PRF would not result in a statistically significant improvement in the socket healing of third molars compared to PRF alone.

## 2. Material and Methods

### 2.1. Study Design

This prospective randomized controlled split-mouth clinical trial was performed in the clinic for oral, maxillofacial and plastic facial surgery of Goethe University in Frankfurt, Germany, having received IBR approval (2024-1701) from the dedicated ethics committee (Ethics committee of the medical department, Goethe University, Frankfurt am Main, Germany; Approval Date: 4 June 2024). Our study was conducted in accordance with the Declaration of Helsinki and relevant ethical guidelines.

### 2.2. Sample Size Calculation

Sample size was calculated at the Institute of Biostatistics and Mathematical Modeling, Goethe University Frankfurt. Statistical power analysis was conducted according to established standards for significance and power. The parameters were set as follows: a type II error (β) of 10% (corresponding to a power of 90%) and a type I error (α) of 5%. The expected minimum detectable difference between groups was estimated using endpoint swelling measurements derived from previously collected pilot study data. The analysis indicated that a minimum of 15 subjects per group would be required to achieve the desired statistical power.

### 2.3. Inclusion and Exclusion Criteria

#### 2.3.1. Inclusion Criteria

a. Patient aged 16 years or older.
b. Indication for Surgery under general anesthesia
c. Patients with bilaterally impacted or partially erupted mandibular third molars requiring surgical extraction.
d. Written informed consent provided.
e. Symmetrical third molars (i.e., similar angulation and position on both sides).
f. Willingness and ability to attend follow-up visits and undergo cone beam computed tomography (CBCT) imaging.

#### 2.3.2. Exclusion Criteria

a. Patient under 16 years of age.
b. No written informed consent provided.
c. Systemic diseases or conditions that may affect wound healing or bone regeneration, such as those requiring immunosuppressive or antiresorptive therapy.
d. Poor patient compliance or inability to adhere to follow-up schedule.
e. Presence of fewer than four third molars.
f. Asymmetrical third molars, i.e., significant differences in angulation or position between the left and right sides.
g. Pregnancy.
h. Patients with allergies to the applied materials.

### 2.4. Surgical Procedure

Presurgical planning included a CBCT scan or orthopantomogram (OPG) to assess the morphology and position of the indicated teeth.

Solid PRF was prepared using patients own peripheral blood. Six tubes of 10 mL (A-PRF, Process for PRF, Nice, France) were collected from each patients before surgery and immediately centrifuged using a table top centrifuge (PRF Duo quattro, Process for PRF, Nice, France). The centrifugation applied 1300 rpm for 14 min. Six clots were isolated, 4 were pressed to generate PRF-plugs and were used for the PRF-side (2 plugs for each socket). The left 2 clots were further processed and mixed with the Bone substitute material (Geistlich Bio-Oss—Geistlich Pharma AG, Wolhusen, Switzerland; 1g) to create sticky bone.

Tooth extractions were performed under general anesthesia by experienced oral surgeons using a minimally invasive approach and conventional osteotomy techniques. All surgeries were performed by the same surgeon to standardized the surgical procedure. Patients were blinded to the treatment allocation. All patients received extraction of bilateral third molars (4 teeth in total: 2 upper and 2 lower molars). Randomization was applied in a split-mouth design. After extraction, the extraction sockets were filled on one side (upper and lower molar) with PRF (2 plugs, 1 for each socket) and on the other side with PRF + BSM. Extraction sites received primary wound closure (suture Novosyn 3-0). Postoperative antibiotic therapy was prescribed (Amoxiclav 875/125 mg, 3×/day for 7 days) and patients were allowed to take analgesics (non-steroidal anti-inflammatory drugs: Ibuprofen 600 mg; max. 3×/day) as needed. Wound healing and pain assessment were monitored over a 7-day period, specifically on postoperative days 1, 2, 3, and 7. Six months after surgery, patients underwent a CBCT scan for assessment.

### 2.5. Postoperative Pain Assessment

Pain was assessed by means of a visual analog scale (VAS). Patients were asked to record their pain at the same time for each side separately on days 1, 2, 3 and 7 after extraction.

### 2.6. Swelling Assessment

Swelling was recorded via distance measurements of specific anatomic regions. In total, three distances were recorded as previously described [19]: distance I (tragus to menton), distance II (tragus to labial commissure) and distance III (gonio angle to eye corner). These were recorded for each side by an independent and blinded physician on days 1, 2, 3 and 7 after extraction.

### 2.7. Radiological Analysis

CBCT scans or panoramic x-rays were performed before surgery to assess teeth angulation and position. After a mean healing period of six months, a CBCT scan was obtained to evaluate bone regeneration.

Radiological visualization and analysis were conducted using the method previously described by Ghanaati et al. [16], with slight modifications. In brief, CBCT data were processed in OsiriX MD (Pixmeo SARL, software version UDI-PI:14.1.1, Bernex, Switzerland) to generate three-dimensional (3D) reconstructions of the jaw, focusing on the region of interest (ROI) including the extraction socket. The threshold was adjusted to visualize both low-mineralized and mature bone. Pseudo-color coding was applied as previously reported [16], with mineralized bone structures highlighted in green and non-mineralized socket regions coded in red. Neighboring anatomical structures were visualized in blue. Representative 3D images of the central section of the alveoli were then analyzed using the ImageJ software 1.45g to quantify the proportion of mineralized and non-mineralized tissue within the ROI in relation to the total socket volume.

### 2.8. Statistical Analysis

Based on the analyzed representative images from the original CBCT, the area from the overlay image was measured in mm^2^. To directly compare any changes, the relative areas of the individual structures (mineralized and non-mineralized bone) were then calculated for the total socket area in percent. Finally, the mean values ± standard deviations/standard error were calculated for each time point. Additionally, numeric data for the swelling and VAS measurements were used. Statistical analysis was performed using a two-way analysis of variance, at an alpha of 0.05, paired with a post hoc test for multiple comparisons (uncorrected Fishers’ Least Significant Difference test) using the GraphPad Prism statistical software (version 10.1.1).

## 3. Results

### 3.1. Patients

**Figure 1:**
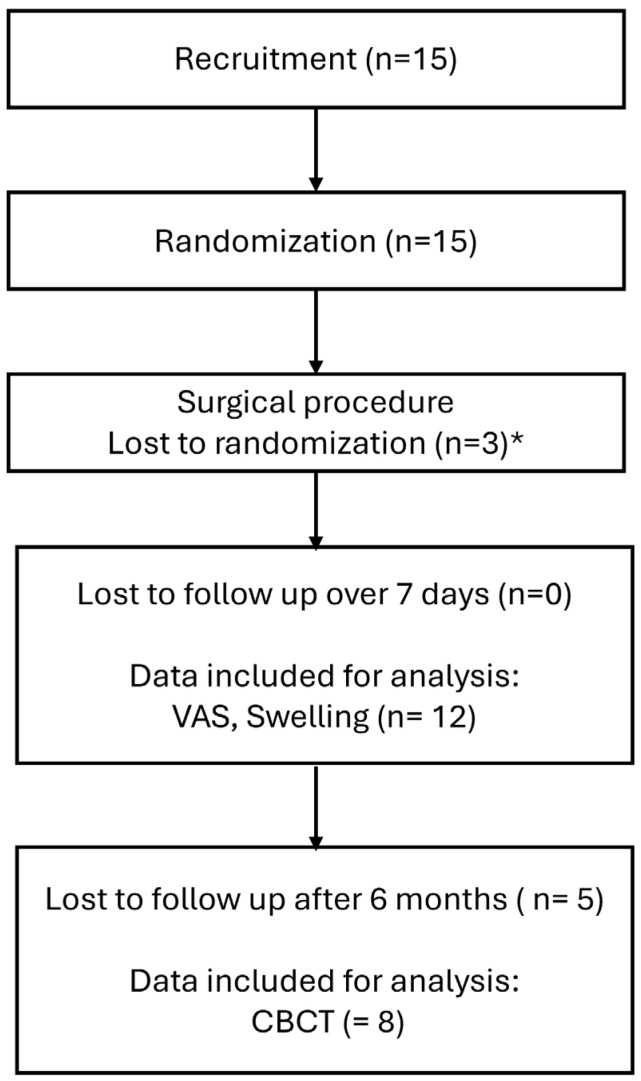
Flowchart of patient recruitment, allocation, and follow-up. * surgical issues preventing augmentation such as antro-oral communication.

Fifteen patients (nine male and six female) with a mean age of 23.6 years were enrolled in this study. Three patients were lost during randomization due to oroantral fistula and thus limitations in the use of bone substitute materials. Twelve patients completed the observation period of 7 days. However, a further five were lost to follow-up after 6 months (Figure 1).

Initial wound healing was uneventful in both groups, with no acute or delayed onset infections observed at any time point. The clinical and radiological parameters we evaluated are presented in the following sections.

### 3.2. Swelling Assessment

**Figure 2:**
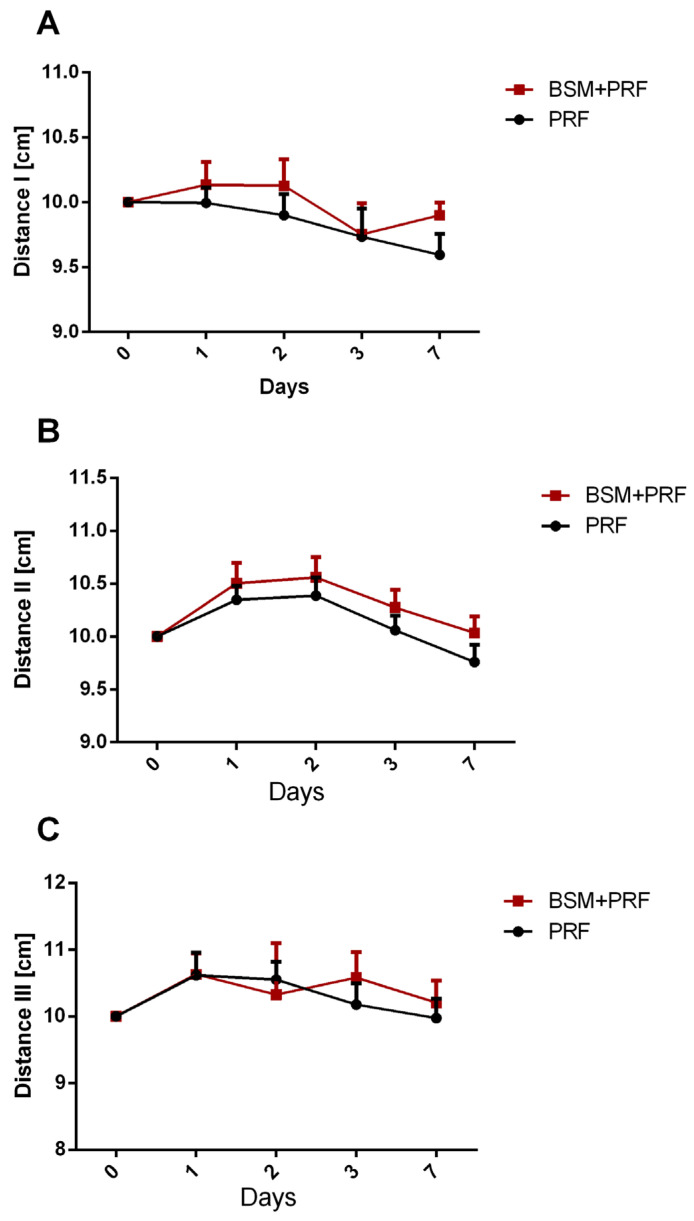
Swelling assessment according to anatomic measurements presented as mean value ± standard error, showing changes in (**A**) distance I (tragus to menton), (**B**) distance II (tragus to labial commissure), and (**C**) distance III (gonio angle to eye corner) for both groups over 7 days. PRF: platelet-rich-fibrin; BSM: bone substitute material.

Swelling was assessed clinically and quantified by measuring three distances in cm. Distance I (tragus to menton) showed a mean value of 10 cm on day 1, and did not show any remarkable changes over the 7 postoperative days. There were no significant differences over time or between the test and control groups (Figure 2A). Distance II (tragus to labial commissure) showed the largest distance increase, reflecting the maximum swelling after 1–2 days postoperatively (10.5 cm). This pattern was observed similarly in both groups. The distance decreased gradually towards day 7, reaching the same level as day 0 of about 10 cm. No statistically significant differences were observed between the test and control groups at any time point (Figure 2B). Distance III (gonio angle to eye corner) also showed increasing distance, reaching a maximum after 1–2 days (10.6 cm) in both groups. The PRF group showed a more rapid decrease in the distance towards day 7 compared to the BSM + PRF group. However, no significant difference between the groups was shown at any time point (Figure 2C).

### 3.3. Pain Assessment

**Figure 3:**
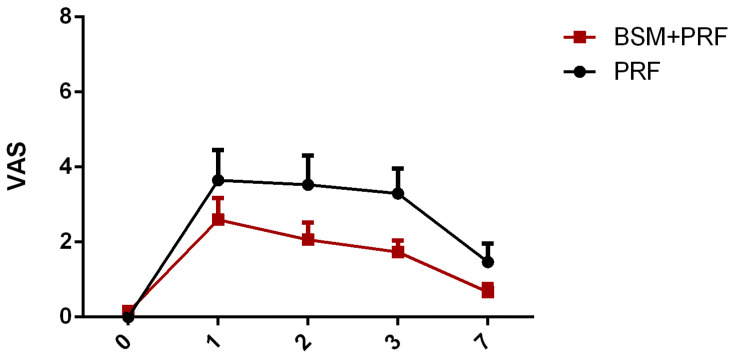
Pain assessment (mean value ± standard error) using a visual analog scale (VAS) over 7 days. PRF: platelet-rich-fibrin; BSM: bone substitute material.

Subjective postoperative pain assessment was measured by means of a visual analog scale (VAS). In both groups, the mean pain was the highest on the first and second postoperative days while, after three days, pain subsequently reduced. The PRF group showed a relatively higher pain score compared to the BSM + PRF group. No statistically significant differences were observed between the test and control groups at any time point (Figure 3).

### 3.4. Radiological Results

**Figure 4:**
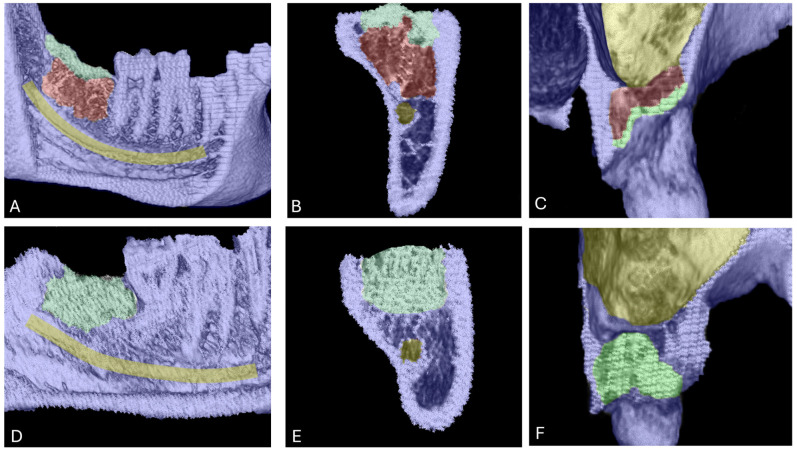
Bone regeneration and morphological changes within extraction sockets. Panels (**A**–**C**) show covered socket residuum (indicated in red) within sockets treated with platelet-rich fibrin (PRF) in the lower (**A**,**B**) and upper jaw (**C**). Mineralized bone areas are highlighted in green and are predominantly located in the coronal portion of the socket. Panels (**D**–**F**) illustrate the regeneration pattern in the group treated with bone substitute material (BSM) combined with PRF. In this group, green coloration indicates homogeneously mineralized bone tissue throughout the socket, with no evidence of covered socket residuum.

New bone regeneration was morphologically evaluated by means of 3D images generated from CBCT data. In the control group (PRF alone), the bony socket healing was not fully regenerated after six months. Regeneration was observed in the uppermost part of the socket, however covered socket residuum were detected in the lower part (Figure 4A–C). This phenomenon manifested as a non-mineralized hole within the socket area that was surrounded by partially newly built mineralized bone (Figure 4A–C). By contrast, the test group (BSM + PRF) showed a fully mineralized socket in most cases. No covered socket residuum was observed in the test group after six months (Figure 4D–F).

**Figure 5:**
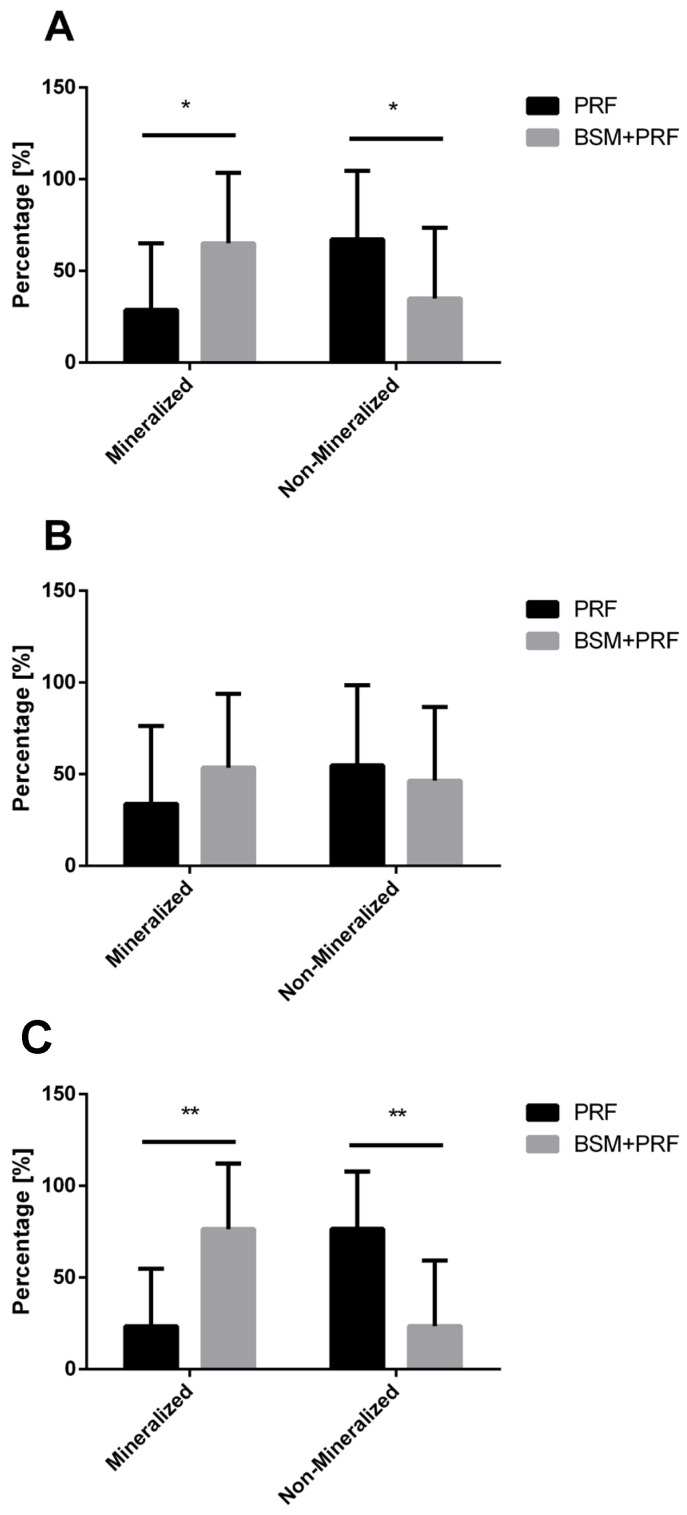
Percentage quantification of mineralized and non-mineralized areas in both groups (mean value ± standard deviation). (**A**) Overall analysis including maxillary and mandibular third molars, * (*p* < 0.05). (**B**) Subgroup analysis of maxillary third molars. (**C**) Subgroup analysis of mandibular third molars. A statistically significant difference was observed between the PRF and BSM + PRF groups in terms of mineralized and non-mineralized tissue percentage ** (*p* < 0.01).

The quantified percentage of the socket region revealed a consistent pattern. When analyzing upper and lower third molars together, the BSM + PRF group exhibited a significantly greater area of mineralized bone compared to the PRF group alone (*p* < 0.05) (Figure 5A). Conversely, the mineralized bone area was significantly reduced in the PRF group relative to BSM + PRF (*p* < 0.05).

A subgroup analysis of upper and lower third molars separately supported this overall trend. For upper molars, the BSM + PRF group showed a higher mineralized bone area and a correspondingly lower non-mineralized area compared with PRF alone; however, these differences did not reach statistical significance and were associated with high variability (Figure 5B).

In contrast, the analysis of lower third molars demonstrated a clearer effect. The BSM + PRF group showed a statistically significantly larger mineralized bone area compared to PRF alone (*p* < 0.01). Conversely, the PRF group exhibited a significantly greater non-mineralized bone area compared to BSM + PRF (*p* < 0.01) (Figure 5C).

## 4. Discussion

Third molar extraction is a common procedure in oral and maxillofacial surgery, but is often associated with intra- and postoperative challenges [1]. Therefore, effective planning and careful execution are just as important as optimal postoperative care in promoting wound healing and regeneration. In particular, good wound healing and bone regeneration after third molar extraction may prevent future complications such as infection, chronic pain, or even osteomyelitis. Therefore, the aim of the present randomized controlled split-mouth clinical study was to evaluate the use of platelet-rich fibrin (PRF)—alone or in combination with bone substitute materials (BSMs)—in supporting socket regeneration and wound healing after third molar extraction.

Various studies have analyzed the role of PRF in promoting wound healing and improving postoperative symptoms following third molar extraction [20,21]. A systematic review of 21 clinical trials assessed PRF application after mandibular third molar removal compared to control treatments. The meta-analysis-based results indicated that PRF effectively reduced postoperative pain, swelling, trismus, and even otitis. However, no clear evidence was found regarding its effect on bone regeneration [22]. Comparable results have been reported in numerous other clinical studies [20,23,24,25]. Similarly, our results showed no significant differences between the two evaluated groups in terms of postoperative swelling and pain. It is important to note that this study employed a split-mouth design, with PRF applied on both sides. Therefore, conclusions regarding the specific efficacy of PRF in reducing swelling or pain cannot be drawn. However, it is noteworthy that, in the test group (BSM + PRF), postoperative symptoms—such as swelling and pain—were not significantly different from those observed in the control group (PRF only). This suggests that the addition of bone substitute material (BSM) did not increase postoperative burden on patients when used in combination with PRF. The pain-reducing effect of PRF can be considered an additional benefit in the context of postoperative regeneration. However, PRF is neither comparable to nor a replacement for conventional analgesic medication. Additionally, PRF’s mechanism of action is complex, involves various components such as inflammatory cells and growth factors, and is not yet fully understood.

In addition, clinically evaluated postoperative wound healing was uneventful in both groups. In keeping with previously reported results, this finding further highlights the role of PRF in supporting wound healing and soft tissue regeneration [14,25,26].

Moreover, the bone regeneration process following third molar extraction is of great importance for maintaining long-term dental health, particularly for the adjacent second molar. Bone regeneration after tooth extraction has been described as a physiological healing process [27]. After tooth removal, the bony defect is initially filled with blood, which coagulates to form a provisional matrix, marking the first phase of healing (hemostasis and coagulation). This is followed by the recruitment of inflammatory cells to initiate the bone regeneration process (inflammatory phase). Subsequently, osteoblasts and other progenitor cells proliferate and begin producing an extracellular matrix to fill the defect with new bone (proliferative phase), which is later remodeled into mature bone (remodeling phase) [28]. However, during this sequence, bone resorption leads to dimensional changes in the alveolar ridge and eventual bone atrophy. This loss of bone volume has been observed to progress most rapidly within the first three months post-extraction and may continue for up to twelve months [27]. Therefore, various techniques have been introduced to prevent bone atrophy and promote bone regeneration after tooth extraction; among these is the application of BSMs. This approach is primarily used to preserve alveolar bone volume for future dental implant placement [29,30]. Although replacing third molars with implants is generally not indicated, maintaining healthy bone volume and supporting regeneration in the extraction site remains essential in preserving the surrounding structures. Thus, insufficient retromolar bone regeneration can lead to chronic inflammation, periodontal breakdown of the distal second molar [31] and other chronic symptoms.

The results of the present study revealed a noteworthy bone regeneration pattern. In the PRF alone group, the coronal part of the socket was predominantly ossified, while the apical part remained occupied by non-mineralized tissue, suggesting the formation of covered socket residuum within the jaw. In contrast, the BSM + PRF group exhibited more extensive mineralization, with homogeneously formed bone throughout the socket. Notably, no covered socket residuum was observed in this group.

Quantitative analysis demonstrated a statistically significant higher percentage of mineralized bone in the BSM + PRF group compared to the PRF alone group. These findings were observable in the overall analysis and more pronounced in the subgroup analysis focusing on lower third molars. The results we present here were only possible to obtain through the use of three-dimensional radiographic imaging and a specific methodology. These findings are consistent with a previous study, which reported a similar phenomenon in unassisted premolar extraction site healing [16]. In that study, such covered socket residuum formation was observed in nearly all cases and was referred to cavitation. These findings were characterized by non-mineralized areas in the apical part of the socket surrounded by limited newly formed bone. Therefore, the results we present here indicate that similar to unassisted wound healing, covered socket residuum were evident within extraction sockets despite treatment with PRF alone [16]. No studies were found that described a three-dimensional analysis of the regenerative process of third molar sockets. Therefore, to the best of our knowledge, this study is the first to describe the covered socket residuum (CSR) observed in the former sockets of third molar, despite PRF treatment. To date, the clinical relevance of this phenomenon—apart from the associated bone deficiency—is not yet fully understood. However, its presence could potentially compromise long-term bone health, support chronic infections and osteomyelitis, and impact the periodontal health of adjacent teeth, particularly the second molar.

A relevant clinical study analyzed tissue samples from the retromolar region of former third molar extraction sites; these sites had been clinically described as showing fatty degeneration and necrotic bone changes. In vitro analysis revealed a 30-fold mean overexpression of Regulated on Activation, Normal T-cell Expressed and Secreted and a 20-fold increase in FGF-2 expression compared to healthy controls [32,33]. These findings may suggest a potential association with the covered socket residuum observed in this study. However, further research is needed to examine the clinical relevance of such covered socket residuum and their potential risk and treatment indications in the long term.

The present study showed that the use of BSMs may prevent covered socket residuum. BSMs are widely used in oral and maxillofacial surgery, providing safe and effective procedures for preventing bone atrophy and supporting bone regeneration [34]. There are different types of BSMs available, including non-resorbable and rapid and slow resorption types. Additionally, different BSM origins are applicable, such as autogenous, allogeneous, xenogenous and others. When looking at the literature, different studies have reported on the evaluation of BSMs in third molar sockets [35,36,37]. However, to date, the most suitable type of BSM for supporting bone regeneration in this specific area remains unknown; more clinical studies are need to answer this important clinical question.

The present randomized controlled split-mouth clinical study yielded clinically and scientifically relevant findings. However, several limitations should be acknowledged. Firstly, due to the split-mouth design, only two groups could be compared. Including a third group treated with BSM alone would have been beneficial in more clearly assessing the clinical efficacy of PRF in combination with BSM. Secondly, our sample size was limited and the number of patients lost during randomization and follow-up, which was larger than initially planned. This was due to patients incompliance during the follow up period as well intraoperative situations (the appearance of oroantral communication in the upper jaw preventing the randomization in the BSM + PRF group). Therefore, there is a need for larger-scale clinical trials to further validate these findings and serve as proof of concept. Additionally, although the three-dimensional radiological analysis used in this study provided valuable insight into bone formation patterns within the evaluated groups, whether the mineralized structures observed in the BSM + PRF group represent newly formed bone or residual BSM particles remains uncertain. Given the radiodensity similarities between BSM and new bone, the ability to distinguish between the two using imaging alone is limited. A histological analysis of the regenerated sites would have been highly beneficial for confirming the nature of the mineralized tissue. However, in the absence of a clinical indication for re-entry surgery, obtaining tissue samples from the regenerated area is difficult to justify, both ethically and practically. Moreover, histological analysis provides only two-dimensional and localized information from specific regions of interest, and therefore is not suitable for fully characterizing three-dimensional phenomena such as those observed in the present study through radiological three-dimensional analysis. We believe that a combined approach including both histological and radiological methods is essential to obtain complementary insights and to achieve a more comprehensive understanding of this complex biological process. Future studies involving other regions of the jaw, where re-entry procedures—such as dental implant placement—allow for the collection of histological samples, are warranted to further validate and correlate the present radiological findings. Additionally, the present study evaluated bone healing at a single time point—six months post-extraction. Long-term follow-up would thus be necessary in order to further characterize the persistence, remodeling, or resolution of the covered socket residuum observed in this study.

Altogether, within the limitations of the present study, our findings allow us to draw some clinically relevant conclusions. Firstly, the use of PRF alone in third molar extraction sites appears to be associated with covered socket residuum formation, whereas the additional application of bone substitute materials (BSMs) supports more homogeneous bone regeneration. Secondly, combining BSMs with PRF results in similar levels of postoperative swelling and pain compared to the use of PRF alone, indicating no additional patient burden when BSMs are used. These findings suggest that patients may profit from the use of PRF to reduce pain and swelling, especially when combined with BSMs. Additionally, the application of BSMs in combination with PRF seem to support bone regeneration and prevent CSR, which may influence the patients systemic health on the long term.

The present study provided key insights about the socket regeneration after third molar extraction. However, further research questions such as the identification of the most suitable BSMs for this indication, evaluating the needed covering techniques in comparison to open wound healing and the role of PRF as an adjuvant biological materials are yet to be analyzed. Additionally, further long term studies are highly needed to outline the effect of CSR on the general patients health and the local bone health.

Based on these findings, we recommend combining BSMs with PRF to support bone regeneration following third molar extraction. However, further research is needed to clarify the clinical significance and long-term consequences of the covered socket residuum observed in PRF-alone cases.

## 5. Conclusions

The present randomized controlled split-mouth clinical trial compared the combined use of BSM and PRF (n = 16) to that of PRF alone (n = 16) in supporting bone regeneration after third molar extraction. Postoperative pain and swelling were assessed over 7 days using the VAS and distance measurements, respectively. New bone formation was evaluated after 6 months using three-dimensional CBCT imaging. No statistically significant differences were found between groups in terms of postoperative pain or swelling at any time point. However, bone regeneration patterns differed notably: the PRF alone group exhibited covered socket residuum in nearly all cases, characterized by mineralization limited to the coronal socket region and non-mineralized tissue beneath. In contrast, the BSM + PRF group demonstrated homogeneous and more complete mineralized bone formation throughout the socket.

These findings highlight the clinical relevance of combining BSM with PRF to enhance bone regeneration following third molar extraction, suggesting that BSM use may help to prevent the formation of covered socket residuum during healing. More studies are needed to outline the most suitable BSM type in this regard.

## Data Availability

The raw data supporting the conclusions of this article will be made available by the authors on request.
